# The Roles of Helix I and Strand 5A in the Folding, Function and Misfolding of α_1_-Antitrypsin

**DOI:** 10.1371/journal.pone.0054766

**Published:** 2013-01-29

**Authors:** Anja S. Knaupp, Shani Keleher, Li Yang, Weiwen Dai, Stephen P. Bottomley, Mary C. Pearce

**Affiliations:** Department of Biochemistry and Molecular Biology, Monash University, Victoria, Australia; Cambridge Institute for Medical Research, United Kingdom

## Abstract

α_1_-Antitrypsin, the archetypal member of the serpin superfamily, is a metastable protein prone to polymerization when exposed to stressors such as elevated temperature, low denaturant concentrations or through the presence of deleterious mutations which, in a physiological context, are often associated with disease. Experimental evidence suggests that α_1_-Antitrypsin can polymerize via several alternative mechanisms *in vitro*. In these polymerization mechanisms different parts of the molecule are proposed to undergo conformational change. Both strand 5 and helix I are proposed to adopt different conformations when forming the various polymers, and possess a number of highly conserved residues however their role in the folding and misfolding of α_1_-Antitrypsin has never been examined. We have therefore created a range of α_1_Antitypsin variants in order to explore the role of these conserved residues in serpin folding, misfolding, stability and function. Our data suggest that key residues in helix I mediate efficient folding from the folding intermediate and residues in strand 5A ensure native state stability in order to prevent misfolding. Additionally, our data indicate that helix I is involved in the inhibitory process and that both structural elements undergo differing conformational rearrangements during unfolding and misfolding. These findings suggest that the ability of α_1_-Antitrypsin to adopt different types of polymers under different denaturing conditions may be due to subtle conformational differences in the transiently populated structures adopted prior to the I and M* states.

## Introduction

Protein misfolding is associated with a range of diseases and occurs when a protein meanders from its normal folding pathway, resulting in the formation of a non-native state that can self-associate. The serpin (serine proteinase inhibitor) superfamily is commonly associated with misfolding and disease. Several members of this superfamily are prone to self-association which is linked to a range of diverse disorders including emphysema, liver disease, angioedema, neurodegeneration and thrombosis [1]. The serpins are particularly susceptible to misfolding and aggregation as they are metastable. The inherent tension within the native state is necessary for protease inhibition, however, mutations can easily result in conformational rearrangements which lead to polymeric states with significantly increased stability [2].

α_1_-Antitrypsin (α_1_AT) is the serpin most commonly associated with misfolding and aggregation as there are several naturally occurring variants linked to disease. The Z variant of α_1_AT carries a Glu to Lys substitution at position 342 and causes disease in approximately one in 2000 individuals [3]. Studies of the un/folding pathways of wild type α_1_AT [4], [5], [6], [7], [8], [9], and Z α_1_AT [10], [11], [12], have indicated that both proteins follow a three-state pathway with the formation of a single intermediate ensemble (I) readily populated in approximately 1 M guanidine hydrochloride (GdnHCl). There is evidence that this folding intermediate of α_1_AT is prone to polymerization [13], [14], [15] and that the Z mutation leads to retardation of the second folding transition and an increased rate of unfolding from the native state and hence accumulation of the aggregation-prone species [11], [12].

Besides low denaturant concentrations other mildly denaturing conditions such as elevated temperatures [15], [16], [17] and low pH [18] can induce α_1_AT polymerization. Extensive biophysical studies into the polymerization pathway of α_1_AT and other serpins have shown that a conformational change to a non-native species occurs prior to the formation of inter-molecular linkages [16], [17], [18], [19], [20] and based on the heat-induced polymerization reaction of α_1_AT this polymerogenic species has been termed M* [16].

Several mechanisms of α_1_AT polymerization have been proposed, and are supported by a range of biochemical data. While all models of α_1_AT polymers involve insertion of the reactive centre loop (RCL) into the A β-sheet (inter- or intra-molecularly), differences have been proposed for other elements of secondary structure that either swap between molecules, or rearrange to accommodate formation of the linkage [3], [21], [22]. The first model, the loop A sheet mechanism, suggests little else besides RCL insertion is required to link protomers in the chain. Recent molecular modeling, however, suggests this seems energetically unfavorable [23]. Two recent crystal structures have demonstrated that other linkages are possible: the C-terminal swap mechanism requires exchange of the whole C-terminus of one molecule with that of another, following self-insertion of the RCL [22], while the strand 4A(s4A)/strand 5A (s5A) mechanism results in the RCL and s5A forming a hairpin which inserts into a neighboring molecule. For this to be achieved other regions of the protein, such as the helix I (hI), must unfold [21]. One of the major determining factors behind the formation of one polymer form over another appears to be the solution conditions under which the polymers are formed. Partial denaturation of α_1_AT in denaturant (which induces formation of the α_1_AT folding intermediate) appears to favor formation of s4A/s5A swap polymer, while heat induced aggregation favors formation of the C-terminally swapped polymers [21], [22].

Improving our understanding of the conformational changes that occur within α_1_AT during polymerization, and during folding, is of critical importance. Our previous work demonstrated that helix F, which lies across the face of the A β-sheet, responds differently to thermal and chemical denaturation, and is thought to therefore sample different conformations when forming either the folding intermediate, I, or the polymerogenic intermediate, M* [24]. The two most recently proposed α_1_AT polymer models suggest that there are other regions which may also adopt different conformations during formation of either type of polymer, in particular: s5A and hI. In this study we have taken an extensive protein engineering approach to probe the behavior of these two structural elements during the early conformational changes that occur during unfolding, and misfolding, and have also identified some interesting behavior associated with the function of α_1_AT as a protease inhibitor.

## Results

**Figure 1:**
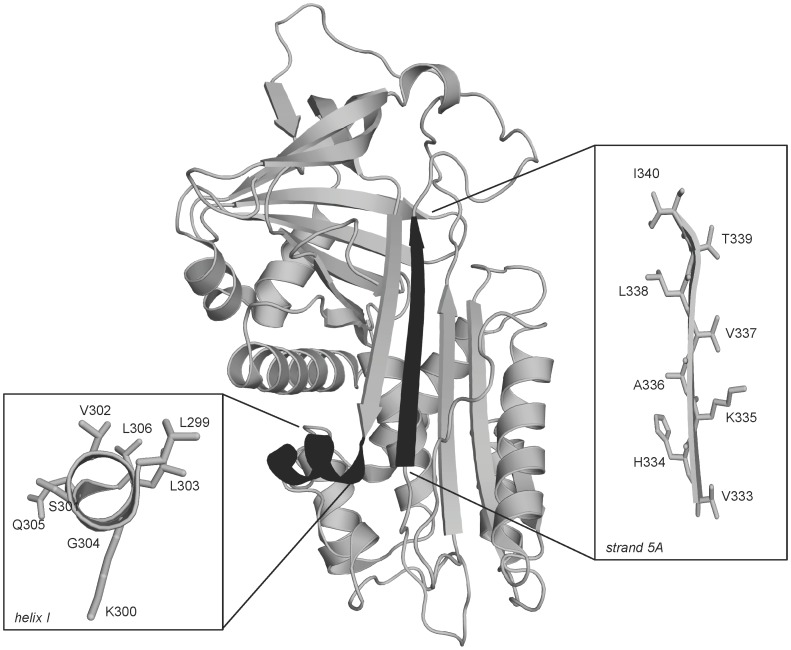
Schematic representation of α_1_AT. Ribbon diagram of α_1_AT (Protein Data Bank ID: 1QLP) with hI and s5A shown in black. The insets show a close-up view of hI and s5A with their residue side chains shown as sticks. Mutations at positions 299, 301, 305 and 334 were not characterized in this study.

Two key structural elements of α_1_AT were selected for investigation in this study: hI and s5A (Figure 1). Individual residues were replaced with a residue that removes a native state contact but is predicted not to alter the local secondary structure around the site of mutation [25]. Twelve α_1_AT variants were expressed in a soluble form using our yeast expression system [26]; five carrying mutations in hI (K300A, V302A, L303A, G304A and L306A) and seven in s5A (V333A, K335A, A336G, V337A, L338A, T339S and I340V). Many of these residues show relatively high sequence conservation across the serpin superfamily (Table 1). A number of variants containing mutations in hI (L299A, S301A, Q305E) and s5A (H334Q) could not be purified in an active monomeric form and were therefore not further analyzed. All α_1_AT variants that were further investigated were monomeric and possessed wild type secondary structure as assessed by far-UV circular dichroism (CD) (data not shown).

**Table 1:** Inhibitory properties of the hI and s5A α_1_AT variants.

	SI	*k*_assapp_ a	*k*_ass_ b	Conservation^c^
		(×10^6^ M^−1^ s^−1^)	(×10^6^ M^−1^ s^−1^)	(%)
wt	1.0±0.1	1.20±0.11	1.20	
K300A	1.3±0.03	0.89±0.08	1.16	40–50
V302A	1.4±0.03	1.86±0.11	2.6	30–40
L303A	1.7±0.02	1.02±0.05	1.73	90
G304A	1.0±0.01	1.18±0.03	1.18	0–20
L306A	1.6±0.03	0.92±0.09	1.47	60–70
V333A	1.2±0.2	0.54±0.01	0.65	50–60
K335A	1.0±0.0	1.27±0.33	1.27	60–70
A336G	1.2±0.1	0.75±0.01	0.90	60–70
V337A	1.0±0.1	0.96±0.23	0.96	50–60
L338A	1.0±0.0	1.26±0.09	1.26	50–60
T339S	1.0±0.0	0.98±0.06	0.98	0–20
I340V	1.1±0.1	0.87±0.07	0.96	60–70

a Calculated using the total concentration of α_1_AT.

b Calculated using the fractional concentration of α_1_AT that forms an inhibitory complex with the proteinase.

c Taken from the genomic analysis performed by Irving *et al.* [34]. Each experiment was performed three times. Errors included represent the standard deviation of each dataset.

### Effects of Mutations in hI and s5A on Protease Inhibition

The stoichiometry of inhibition (SI) and the association rate constant (*k*_ass_) of each variant were determined against bovine chymotrypsin and showed that all mutants were functional protease inhibitors (Table 1). The *k*_ass_ values of all mutants were similar to the wild type value of 1.2±0.11 ×10^6^ M^−1^ s^−1^. The SI values of all s5A mutants were within error of the value of wild type α_1_AT (SI = 1.0±0.1). However the mutations within hI had a significant effect upon the SI. Replacement of K300, V302, L303 and L306 with an alanine residue resulted in elevated SI values for all four proteins (Table 1).

### Effects of Mutations in hI and s5A on Native State Stability and Equilibrium Unfolding

**Figure 2:**
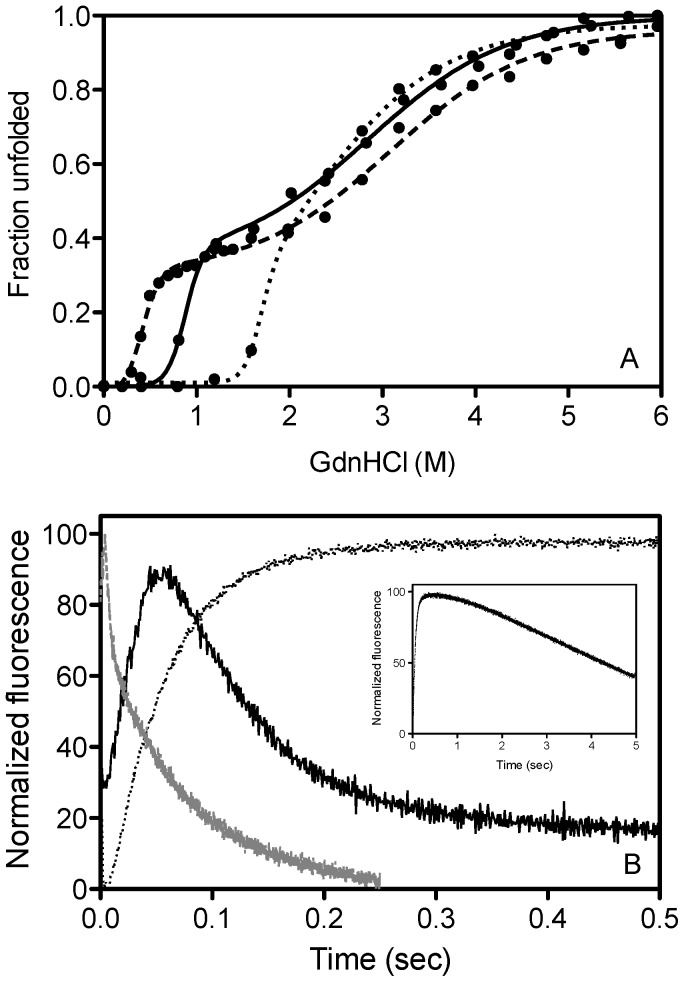
Representative traces for the unfolding reactions of wild type α_1_AT and the variants which showed the most significant difference. (a) GdnHCl-induced equilibrium unfolding curves of wild type α_1_AT (solid line), the V333A variant (dashed line) and the K335A variant (dotted line). Data were obtained by monitoring the change in CD signal at 222 nm, and represent the average of three separate experiments, while lines represent three-state curve fits. (b) Kinetic traces of α_1_AT variants unfolding in 4 M GdnHCl. Wild type α_1_AT is shown in solid black, the K335A variant in dotted black and the V333A variant in gray. The inset depicts the trace for the K555A variant over a longer time period. Traces were obtained by monitoring the change in bis-ANS fluorescence above 450 nm, and represent the best of 10 experiments. Data were fit to two separate single exponential equations, and analyzed in the Proviewer package supplied with the instrument.

Thermal melts and GdnHCl-induced equilibrium unfolding studies using far-UV CD were performed in order to obtain information on the role of hI and s5A residues in maintaining native state stability (Table 2, Figure 2a). The equilibrium unfolding reaction of α_1_AT has been studied extensively and is defined as a reversible three-state transition with an intermediate ensemble (I) populated between the native (N) and the unfolded (U) state [4], [5], [7], [8], [27]. All α_1_AT variants analyzed unfolded via a similar three-state unfolding reaction. For wild type α_1_AT we observed two transitions with midpoints of 0.86 M GdnHCl for the first transition (N→I) and 2.93 M GdnHCl for the second transition (I→U). None of the mutations altered the I→U transition significantly, however, several hI and all s5A variants displayed an altered midpoint of the N→I transition.

**Table 2:** Kinetic and thermodynamic stability properties of the hI and s5A α_1_AT variants.

	*k*_N→I_ a	*k*_I→U_ a	*D*_mN→I_ b	ΔΔ*G*_N→I_ b	*D*_mI→U_ b	ΔΔ*G*_I→U_ b	*T*_m_ c
	(s^−1^)	(s^−1^)	(M)	(kcal/mol)	(M)	(kcal/mol)	(°C)
wt	55.62±1.0	10.37±0.04	0.86±0.02	–	2.93±0.15	–	62.4±1.2
K300A	43.04±0.4	11.11±0.01	0.62±0.09	1.46	2.93±0.01	0.00	63.0±0.5
V302A	54.93±1.7	9.72±0.03	0.83±0.05	0.18	3.04±0.24	0.09	61.7±0.5
L303A	130.5±2.2	15.68±0.02	0.85±0.03	0.06	2.99±0.07	0.05	59.9±1.0
G304A	81.25±1.2	19.6±0.03	0.71±0.12	0.92	2.83±0.02	0.09	61.9±0.4
L306A	58.77±0.6	17.09±0.03	0.63±0.01	1.40	3.01±0.09	0.06	61.3±0.6
V333A	N/A	13.75±0.02	0.46±0.06	2.44	3.15±0.11	0.18	55.0±0.4
K335A	20.3±0.1	0.03±0.0	1.75±0.07	5.43	2.24±0.27	0.55	76.7±0.4
A336G	N/A	13.52±0.05	0.49±0.07	2.26	2.91±0.01	0.02	58.2±1.2
V337A	64.37±3.1	13.81±0.12	0.73±0.02	0.79	2.99±0.08	0.05	62.3±0.9
L338A	88.58±1.6	13.42±0.04	0.99±0.07	0.79	2.82±0.08	0.09	65.4±0.7
T339S	62.46±1.2	15.49±0.03	0.77±0.06	0.55	2.79±0.22	0.11	62.9±1.1
I340V	63.2±1.9	14.91±0.03	0.75±0.08	0.67	2.87±0.07	0.05	62.4±0.5

a *k*_N→I_ and *k*_I→U_ represent the rates for the N→I and the I→U transitions when the proteins were denatured in 4 M GdnHCl.

b The GdnHCl-induced unfolding curves were analyzed using the three-state unfolding function to determine the midpoints of the N→I transition (*D*_mN→I_) and the I→U transition (*D*_mI→U_). ΔΔ*G*_N→I_ represents the change in free energy for the N→I transition (all experimental errors were within ±0.12 kcal/mol) and ΔΔ*G*_I→U_ the change in free energy for the I→U transition (all experimental errors were within ±0.12 kcal/mol).

c The thermal denaturation curves obtained using far-UV CD were analyzed using the two-state unfolding function to determine the midpoint of transition. Each experiment was performed three times. Errors included represent the standard deviation of each dataset.

Several of the mutations introduced to hI caused a decrease in the midpoint of the transition from N→I(*D*_mN→I_), however, the degree to which this occurred varied. The hI mutations K300A, G304A and L306A led to a significant lowering in *D*_mN→I_ with values of 0.62, 0.71 and 0.63 M GdnHCl, respectively, (Table 2). Introducing mutations into s5A lead to a variety of effects, as some mutations raised *D*_mN→I_, while others decreased it. While the mutations V337A, T339S and I340V resulted in a small decrease in *D*_mN→I_ by approximately 0.1 M GndHCl, the V333A and A336G mutations resulted in a major decrease by approximately 0.4 M GndHCl (Table 2). Conversely, the s5A mutations K335A and the L338A resulted in an increase in *D*_mN→I_ to 1.75 and 0.99 M GndHCl, respectively. This is in good agreement with the thermal melt data, seen in Table 2, which suggest that the V333A and A336G mutations led to a significant decrease and the K335A and L338A mutations to a significant increase in the midpoint of thermal unfolding (*T*_m_).

### Effects of Mutations in hI and s5A on Kinetics of Unfolding

In order to obtain additional information on the unfolding pathway of α_1_AT and the role of hI and s5A in the transition state adopted between N and I, kinetic analyses of the unfolding reaction of all mutants were carried out using stopped-flow fluorescence (Table 2, Figure 2b). Unfolding into 4 M GdnHCl, which results in complete unfolding of α_1_AT, allowed analysis of both the N→I and I→U transitions for almost all mutants. At this GdnHCl concentration the first unfolding transition of the α_1_AT variants V333A and A336G occurred too fast to be captured, however, contribution of refolding at lower GdnHCl concentrations did not allow us to decrease the denaturant concentration. The rates of the transition from N→I and I→U were calculated and referred to as *k*_N→I_ and *k*_I→U_, respectively (Table 2).

The majority of s5A mutations resulted in acceleration of *k*_N→I_, with the V333A and A336G variants unfolding so quickly that the transitions occurred within the deadtime of the instrument. The mutants V337A, T339S and I340V led to a slight increase in *k*_N→I_ from 55.62 s^−1^ for wild type α_1_AT to 64.73 s^−1^, 62.46 s^−1^ and 63.2 s^−1^, respectively. Interestingly, the K335A mutation in s5A results in a dramatic decrease in *k*_N→I_ to 20.3 s^−1^(Table 2).

Mutation of residues in hI had a varied effect on the rate of unfolding of α_1_AT. In terms of thermodynamic stability, two hI mutants did not show a change in native state stability in comparison to wild type α_1_AT: V302A and L303A (Table 2), however, the thermodynamic behavior of these mutants varied. The V302A mutation did not affect the unfolding kinetics, while the L303A mutation resulted in an increase in *k_N→I_* to 130.5 s^−1^ which suggests that this residue forms interactions in the transition state, the disruption of which leads to faster unfolding. On the other hand, the K300A and L306A mutations resulted in a decrease in the thermodynamic stability but either in no change of *k_N→I_* (L306A) or in a decrease in *k_N→I_* to 43.04 s^−1^ (K300A). Since, despite their decreased thermodynamic stability, neither mutant displayed an increase in *k_N→I_*, these data suggest that both mutations result in an increase in the free energy of the transition state. The G304A mutation resulted in a decrease in the thermodynamic stability concomitant with an increase in *k_N→I_* indicating that this mutation results in a decrease in the activation energy. However, in comparison to the s5A mutants V337A, T339S and I340V, which showed a similar destabilization of the native state, the *k_N→I_* value was not comparable but at least 20% faster for the G304A variant. This suggests that the G304A mutation might not only lead to a decrease in the activation energy but also to a decrease in the free energy of the transition state. Together, the stopped-flow unfolding data therefore indicate that the residues K300, L303, L306, L338 and possibly also G304 form native contacts in the transition state occupied between N and I.

### Effects of Mutations in hI and s5A on the Rate of Polymerization

**Figure 3:**
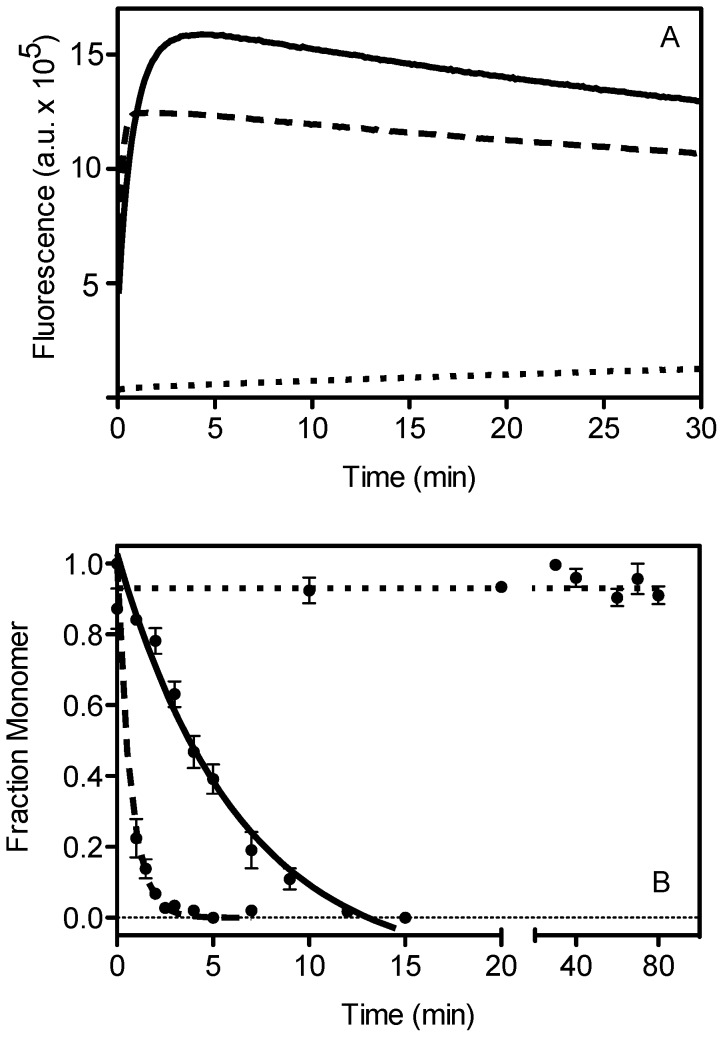
Representative traces for the polymerization reaction of wild type α_1_AT and the variants which showed the most significant difference. Polymerization of the hI and s5A α_1_AT variants was initiated by incubation at 60°C and monitored by a) bis-ANS fluorescence and b) native PAGE. In both graphs the solid line represents wild type α_1_AT, the dashed line represents the V333A variant, and the dotted line represents the K335A variant. Each dataset represents the average of three separate experiments.

Serpin polymerization forms the molecular basis of most serpinopathies, and if induced *in vitro* by heating, occurs via the formation of a non-native monomeric species termed M*, which is highly polymerogenic and self-associates to give rise to long chain polymers [16]. α_1_AT polymers formed at elevated temperature bind the 2C1 antibody which also recognizes Z α_1_AT polymers found in hepatocellular inclusions of patients [28]. As heating α_1_AT results in polymers with pathological features, and M* is adopted prior to polymer formation, it represents an interesting structure that could be targeted for therapeutics, yet its structure remains unknown due to the challenges associated with studying unstable, partially folded states. Here we have analyzed the thermal polymerization characteristics of our mutants, to gain insight into the structure of M*. The rate of M* formation (*k*_cc_) and subsequent polymerization (*k*_agg_) were determined by following the changes in bis-ANS (4, 4′-dianilino-1, 1′-binaphthyl-5, 5′-disulfonic acid) fluorescence at 60°C (Figure 3a and Table 3). Additionally, native PAGE was performed to follow the rate of polymerization as a function of monomer loss (*k*_agg_) (Figure 3b and Table 3).

**Table 3:** Polymerization properties of the hI and s5A α_1_AT variants.

	*k*_cc_ (× 10^−2^ s^−1^)	*k*_agg_ (× 10^−4^ s^−1^)	*k*_-*mon*_ (*x* 10^−3^ s^−1^)
wt	1.93±0.21	1.03±0.04	3.50±0.20
K300A	3.32±0.22	7.15±0.33	7.24±0.47
V302A	2.02±0.05	2.52±0.29	5.11±0.19
L303A	1.45±0.09	0.75±0.02	3.21±0.20
G304A	2.19±0.14	1.98±0.27	5.87±0.31
L306A	5.48±0.11	3.23±0.15	13.02±0.39
V333A	11.94±0.24	1.95±0.04	23.43±1.03
K335A	0.02±0.00	–	0.01±0.01
A336G	7.50±0.47	1.94±0.17	15.90±0.36
V337A	2.58±0.31	1.37±0.04	5.49±0.35
L338A	0.52±0.07	0.055±0.008	1.69±0.05
T339S	1.73±0.10	3.01±0.03	4.46±0.20
I340V	1.43±0.10	1.51±0.04	3.64±0.19

The polymerization kinetics were determined at 60°C by following the changes in bis-ANS fluorescence at a protein concentration of 1 µM. *k*_cc_ is the rate of conformational change and *k*_agg_ the rate of aggregation. The rate of polymerization at 60°C was additionally determined at a protein concentration of 10 µM as a function of monomer loss (*k_-mon_*) on native gels. Each experiment was performed three times. Errors included represent the standard deviation of each dataset.

It has previously been proposed that any decrease in native state stability would result in an increase in the equilibrium concentration of M* and hence accelerate polymerization [16]. This is in good agreement with our polymerization data as the most destabilizing mutations in hI (K300A and L306A) and s5A (V333A and A336G) also resulted in an increased rate of M* formation and polymerization. Similarly, the stabilizing s5A mutations K335A and L338A led to a decreased rate of conformational change and accordingly slower polymer formation.

## Discussion

α_1_AT polymerization leads to disease in approximately one in 2000 individuals and can be induced *in vitro* by incubation of α_1_AT under mildly denaturing conditions [13], [14], [15], [16], [17], [18]. Heating α_1_AT can induce polymerization via formation of a polymerogenic intermediate, M*, and antibody studies suggest these polymers most closely represent those formed in livers of α_1_AT deficiency patients [29]. Polymers can also form when α_1_AT is incubated under conditions that favor formation of the folding intermediate, I [4], [5], [7], [8], [27], though whether these polymers are formed *in vivo* remains unknown. There is, however, evidence that I is important during polymerization, as the most common pathological α_1_AT variant, Z α_1_AT (E342K), is characterized by a retarded folding transition from I to the native state [12], which is thought to be associated with the retention of this protein in hepatocytes [14].

In this study we generated a range of mutants of α_1_AT to probe the behavior of two other structural elements, hI and s5A, two parts of the α_1_AT native structure proposed to undergo conformational change during formation of at least one of these partially unfolded, intermediate structures [6], [21]. Five hI mutants (K300A, V302A, L303A, G304A and L306A) and seven s5A mutants (V333A, K335A, A336G, V337A, L338A, T339 and I340V) could be purified in a monomeric form and were analyzed.

### The Role of hI and s5A during Protease Inhibition

No mutation studied here affected docking of the protease during inhibition, as indicated by the *k*_ass_ values, however altered SI values indicated that hI and s5A have differing roles during inhibition (Table 1). Data presented here, and by others [30], indicate that mutating residues in s5A does not interfere with RCL insertion during inhibition, suggesting that either this strand undergoes deformation at this stage of inhibition, or individual mutations within the strand are not sufficient to cause disruption to the mechanism of inhibition [31]. Most mutations in hI on the other hand resulted in increased substrate behavior. The only hI mutant which displayed an SI indistinguishable from wild type α_1_AT was the G304A variant which is also the residue with the lowest sequence conservation in hI (Table 1). This suggests that during protease inhibition hI provides structural rigidity at the base of the α_1_AT molecule which ensures efficient RCL insertion.

### The Role of Helix I and s5A in Maintaining Native State Stability

The thermodynamic stability of native α_1_AT is more reliant upon native contacts in s5A than hI. It has previously been proposed that over-packing of the side chain of K335, located within s5A and pointing into the body of the molecule, plays an important role in the metastability and inhibitory function of α_1_AT by mediating the strain at this position necessary for efficient protease inhibition [32]. A mutational approach revealed that the size, polarity and shape of the side chain of residue 335 affects the stability and the inhibitory activity with the smallest residues, glycine and alanine, leading to the most significant increase in stability and yet only a minor decrease in inhibitory activity [33]. This is in good agreement with our work which showed that the K335A substitution resulted in a significant increase in the thermodynamic stability of α_1_AT but no decrease in inhibitory activity against bovine chymotrypsin. Additionally, the replacement of L338 with an alanine residue also led to an increase in native state stability without a concomitant decrease in inhibitory activity.

The most highly conserved residue in s5A is H334 which is conserved in approximately 78% of all serpin sequences [34]. It has previously been shown that the replacement of H334 with either a serine or an alanine residue leads to significant destabilization and increased polymerization of α_1_AT [30]. The critical role of the hydrogen network centered on H334 in preventing polymerization is further supported by our work as we were unable to produce native protein upon mutation of H334 to a glutamine residue. Mutation of two additional s5A residues resulted in a significant decrease in native state stability of α_1_AT: V333 and A336. A336 faces into the body of the molecule and substitution to a glycine destabilized the native state significantly. The A336G mutation reduces the hydrophobicity at this position thereby decreasing hydrophobic interactions which the center of this strand makes with the core of the molecule suggesting A336 has a similarly protective role as H334. V333, which also possesses a small side chain, faces away from the body of the molecule and interacts with the loop linking helix F and strand 3A. Therefore, our work strongly suggests that the N-terminal region of s5A is critical in maintaining native state stability.

### The Role of hI and s5A in Folding, Misfolding and Polymerization

A number of biophysical studies indicate that the α_1_AT intermediate ensemble populated at low denaturant concentrations contains about 80% of the native secondary structure including a well formed β-sheet B, partially formed β-sheets A and C and a non-native helix F [4], [5], [11], [24], [27] and H/D exchange mass spectrometry data suggest that it shares many similarities with a molten globule [35]. More recent studies utilizing limited proteolysis [21] and labeling of free thiols [6] indicate that hI and s5A are unstructured in I. Considering we did not observe any changes in the midpoint of the I to U transition upon mutation of hI and s5A residues (Table 2) our equilibrium unfolding data support these findings. Our work has focused predominantly on the first unfolding transition (N→I), as the second transition is non-cooperative, as seen by the slope of our equilibrium unfolding curves, and work published by others [35], which could mask small changes caused by introduction of mutations.

Analysis of the unfolding kinetics of the various α_1_AT variants suggests that residues in hI form native interactions in the transition state adopted between N and I, while only one position in s5A (L338) makes native contacts (Table 2). The most significant increase in *k*_N→I_ was observed for the hI residue L303 when mutated to an alanine. With approximately 90% sequence conservation amongst the serpin superfamily [34] L303 is the most highly conserved residue investigated in this study and our data suggest that this is because of its role in directing folding and preventing misfolding from I. L338, which is highly conserved at this position in s5A, is the only residue which forms interactions in the transition state between N and I. Considering that mutation of L338 to an alanine resulted in a significant increase in the thermodynamic stability of α_1_AT, the data suggest that this residue might have a protective role during folding, similar to others in the strand such as H334 [30]. In terms of α_1_AT folding, these kinetic data suggest that in the transition state L338 tethers the unstructured chain to the body of the molecule and the remaining s5A residues form native contacts only in the final steps of the folding process. This is in good agreement with recently published H/D exchange mass spectrometry data which indicate that s5A is the last region of the α_1_AT molecule to gain protection during kinetic refolding [36]. Intriguingly we do observe some changes to the rates of unfolding from I to U caused by the introduction of some mutations and the simplest explanation for this is that the loss of those hydrophobic interactions significantly speed up the I to U transition.

It remains unclear how the folding intermediate I and the polymer precursor M* relate, and challenges are associated with extensive characterization of both species. The transition of α_1_AT to either conformation is accompanied by an increase in tryptophan [7], [16] and bis-ANS [10], [17] fluorescence, while both display similar protease digestion patterns [21] and both are able to form polymers [13], [14], [15], [16], [17]. Together, these data suggest that they may share similar structural features, however, the immunological evidence that both polymerization conditions lead to different polymer types [22], [28] suggests there are likely to be differences in the intermediate conformations as well. Previous work has already shown that helix F undergoes different conformational changes during unfolding and heat-induced polymerization [24] and this work now suggests that this is also the case for hI and s5A.

A number of mutations which altered *k*_N→I_ did not have a similar effect on the rate of M* formation (Tables 1 and 2). With the L303A (hI) and the L338A (s5A) mutations increasing and the K300A (hI) mutation decreasing *k*_N→I_ those mutations had an opposing effect on the rate of M* formation relative to the wild type. There might be additional residues with this effect, particularly in hI, however, the effect on the transition state is masked by the decrease in native state stability (e.g. G304A and L306). Together, these data suggest different transition states during the formation of I and M* and that particularly hI undergoes alternative conformational rearrangements. Interestingly, this is the structural element which is exposed in the s4A/s5A swap polymer and in its native conformation in the C-terminal swap polymer [21], [22]. Therefore, our data imply that a divergence of the two differing polymerization pathways occurs very early on in the unfolding and misfolding reactions, supporting the theory that subtle differences may exist in the I and M* states which could guide the formation of one polymer over another.

## Materials and Methods

### Materials

Guanidine hydrochloride (GndHCl) was purchased from Sigma and 4,4′-dianilino-1,1′-binaphthyl-5,5′-disulfonic acid, dipotassium salt (bis-ANS) was obtained from Invitrogen.

### Production of the Recombinant α_1_AT Mutants

The α_1_AT mutants were generated using the pHILD2 vector (Invitrogen) carrying the α_1_AT gene as a template [26]. To introduce a single amino acid substitution the according residues were mutated with KOD DNA polymerase (Novagen) using the Quick-change site directed mutagenesis approach (Stratagene) and all mutations were verified with DNA sequencing. The α_1_AT mutants generated were electroporated into *Pichia pastoris* and expressed and purified as described previously [26]. All variants were assessed for contamination by stable conformations through the use of native PAGE in our polymerization assays (see below), and all were found to polymerize completely, indicating that no latent or cleaved material was present.

### Characterization of the Inhibitory Properties

The stoichiometry of inhibition (SI) and the association rate constant (*k*_ass_) against bovine chymotrypsin and the α_1_AT mutants were determined as described previously [26].

### Thermal Denaturation

Thermal denaturation measurements were performed on a Jasco J-815 circular dichroism (CD) spectrometer at a protein concentration of 0.25 mg/mL in 90 mM NaCl and 50 mM Tris, pH 8.0 using a 0.1-cm path-length quartz cell. A heating rate of 1°C/min was applied and the change in signal at 222 nm in the far-UV CD spectra was determined. To obtain the midpoint of denaturation (*T*_m_) the thermal denaturation data were fit to a two-state unfolding model using a non-linear least-squares fitting algorithm as described previously [37].

### Chemical Denaturation

Equilibrium unfolding curves were obtained by following the change in signal at 222 nm in the far-UV CD spectra as a function of GndHCl concentration, following an incubation time of 2 hrs, as described previously [10]. The equilibrium unfolding data were analyzed using a three-state model that recognizes the presence of one stable intermediate structure (I) during the transition from the folded state (N) to the unfolded state (U) as described previously [7], [10], [27]. ΔΔG values were calculated according to [32].

### Unfolding Kinetics

Unfolding kinetics were determined by following changes in the fluorescence emission of bis-ANS at wavelengths >455 nm using a cutoff filter and an excitation wavelength of 410 nm. Experiments were performed at room temperature on an Applied Photophysics SF.18MV stopped-flow apparatus by rapidly mixing 1 volume of protein solution at a concentration of 10 µM with 10 volumes of concentrated GdnHCl solution at room temperature. Both solutions contained 5 µM bis-ANS, 90 mM NaCl and 50 mM Tris, pH 8.0. Data were fitted to two separate single-exponential functions with a term included for baseline instability using the manufacturer’s software.

### Polymerization Assays

Bis-ANS fluorescence measurements were performed on a ***FluoroMax*** **-** ***4*** spectrofluorometer (HORIBA Jobin Yvon) in a 1-cm path-length quartz cell at 60°C. The fluorescence measurements were performed at a final concentration of 1 µM protein, 5 µM bis-ANS, 90 mM NaCl and 50 mM Tris, pH 8.0. The solution in the cuvette was stirred constantly and the fluorescence data was recorded every 1 s. The excitation wavelength (*λ*_ex_) was 410 nm and the emission wavelength (*λ*_em_) was 480 nm. Excitation and emission slit widths were set at 2 nm. The reaction mixture was pre-warmed to 60°C and the bis-ANS signal stabilized before the protein was added. The polymerization kinetic data was analyzed as described previously [17]. The rate of monomer loss upon polymerization at was determined using continuous native PAGE as described previously [11]. Samples of protein (10 µM) were incubated at 60°C in 90 mM NaCl and 50 mM Tris, pH 8.0, and put on ice at various time points to quench the reaction. Ice-cold non-denaturing sample buffer was added, and samples were separated at 4°C on 6% continuous native gels. The rate of polymerization was determined by following the loss of monomer using an exponential decay function.
